# G2D: a tool for mining genes associated with disease

**DOI:** 10.1186/1471-2156-6-45

**Published:** 2005-08-22

**Authors:** Carolina Perez-Iratxeta, Matthias Wjst, Peer Bork, Miguel A Andrade

**Affiliations:** 1 Ontario Genomics Innovation Centre, Ottawa Health Research Institute. ON K1H 8L6, Ottawa, Canada; 2 Institut für Epidemiologie, GSF Forschungszentrum für Umwelt undGesundheit Ingolstadter, Landstrasse 1 D-85758, Neuherberg/Munich, Germany; 3 European Molecular Biology Laboratory, D-69118 Heidelberg, Germany; 4 Department of Bioinformatics, Max Delbrück Center for Molecular Medicine, Robert-Rössle-Strasse 10, 13092 Berlin, Germany

## Abstract

**Background:**

Human inherited diseases can be associated by genetic linkage with one or more genomic regions. The availability of the complete sequence of the human genome allows examining those locations for an associated gene. We previously developed an algorithm to prioritize genes on a chromosomal region according to their possible relation to an inherited disease using a combination of data mining on biomedical databases and gene sequence analysis.

**Results:**

We have implemented this method as a web application in our site G2D (Genes to Diseases). It allows users to inspect any region of the human genome to find candidate genes related to a genetic disease of their interest. In addition, the G2D server includes pre-computed analyses of candidate genes for 552 linked monogenic diseases without an associated gene, and the analysis of 18 asthma loci.

**Conclusion:**

G2D can be publicly accessed at .

## Background

Mutations in genes are responsible for inherited diseases. The discovery of the association of a gene with a disease is essential for potential diagnosis and treatment and often helps understanding the mechanisms involved. The detection is usually a several step "gene hunt" where the gene is first located within a genomic region by linkage to anonymous markers followed by actual sequencing to find all genetic variation and finally to test for association of gene variants typical in diseased subjects [1].

Because the region eventually linked to a disease might contain hundreds of genes, and genotyping or directly sequencing genes of patients and controls is costly, it is important to use available information such as the complete sequence of the human genome plus a set of annotated genes and their functions (either known or predicted) to target the sequencing effort on those genes that appear to have more chances of being associated with the disease. The key to this prioritization is the expectation of the relation of a gene function to a disease (for example, a defect in a neural receptor could produce a neurological disease).

**Figure 1:**
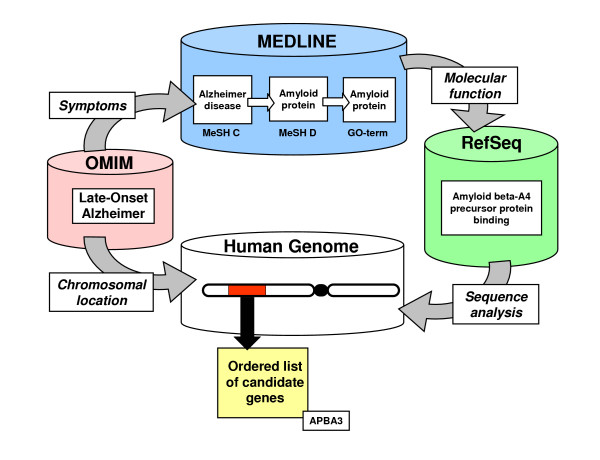
**The G2D algorithm**. The cylinders represent public databases. **MEDLINE **contains references to scientific literature annotated at the National Library of Medicine with terms from the MeSH ontology. For each disease being studied we take the MeSH C terms ('Diseases Category') from the publications associated in **OMIM **[3] as its keywords. For each gene we take the Gene Ontology (GO) terms [8] associated to its product in the **RefSeq **protein database [34] as its keywords. MEDLINE does not contain enough clinical literature to allow us to directly relate every symptom, represented by a MeSH C term, to every gene feature, represented by a GO term. Taking into account that genes relate to phenotypes by means of molecules, we can increase the robustness of the gene/phenotype relations using an intermediate association step through the MeSH D category of 'Chemicals & Drugs' (top). Accordingly, we first compute associations between MeSH C terms ('Diseases') and MeSH D terms ('Chemicals & Drugs') by their co-annotation on the same record, more specifically looking for dependences of MeSH D terms on MeSH C terms. For example, we would deduce a relation between "Alzheimer's disease" (MeSH C) and "Amyloid protein" (MeSH D) if the presence of the C term in a MEDLINE entry always implies the presence of the D term. Records in the RefSeq database contain annotations from GO that describe the protein function, and will often include a link to MEDLINE, mostly dealing with the experimental characterization of the protein. We use these links to relate MeSH D terms from the MEDLINE reference to GO terms from the sequence, again looking for GO term dependence on a MeSH D term. In this case we could deduce an association between the MeSH D term "Amyloid Protein" and the GO term "Amyloid Protein". Finally, we combine both sets of relations to obtain associations between MeSH C terms and GO terms (for example, the relation of Alzheimer's disease to the amyloid protein). To evaluate the genes associated with a particular disease we follow two directions. First, we deduce the gene functions (GO terms) related to the disease using the associations from phenotypes (MeSH C terms) describing the disease. For this, we collect the MeSH C terms found in the MEDLINE references from its corresponding OMIM entry (left), score all GO terms according to their relation to the terms in the MeSH C list (top), and finally, score all the proteins in RefSeq with the average of scores of their GO terms (right). For example, the analysis of late-onset familial Alzheimer disease (LOFAD) [9] would start by characterizing the disease with the MeSH C term "Alzheimer's Disease" among others. This would point to a series of GO terms including "Amyloid Protein" as a likely related function. One of the most related sequences in RefSeq (according to its GO annotations) would be the human amyloid beta A4 precursor protein-binding, which is annotated with the GO-term "amyloid protein". The other component of the analysis is a BLAST homology search [35] of the human genome region where the disease is mapped against the sequences stored in the RefSeq database (bottom). All hits in the region (red block) below a cut-off of E-value of 10e-10 are registered and sorted according to the score of the RefSeq protein they hit. Following our example, the analysis of the region where the LOFAD was mapped would show a gene similar to the human amyloid beta A4 precursor protein-binding annotated with the GO-term "amyloid protein": the APBA3 gene, which interacts with the Alzheimer's beta-amyloid precursor protein [12]. The analysis of LOFAD is extensively described in the Results section. Further details of the method are given in [2] and in the G2D web site.

Following these ideas, we developed an algorithm to relate genes to human inherited diseases that combines the extraction of relations between phenotypes and gene functions in sequence, disease, and literature databases, with sequence similarity searches [2] (Figure 1). The main assumption of this method is that for a given disease with an undiscovered associated gene X, and a phenotypically similar disease with a known associated gene Y, some functions of the X and Y genes will be related and relevant to those phenotypes.

We now implemented this method in the G2D web site allowing users to analyse diseases and genetic regions of their interest. The web site includes a collection of precomputed analyses of 552 inherited monogenic diseases stored in the OMIM database [3] that were linked to a genomic region but not yet associated with a gene. Here we describe the latest update of the method and illustrate its use via the G2D web server to propose original target genes for one monogenic disease and for asthma, a complex disease.

## Implementation

The algorithm needs basically two inputs to work with: a phenotypical definition of a disease as a list of weighted MeSH terms of the C category ('Diseases' category) [4], and the definition of a genomic region in the human genome where it has to search for genes potentially associated with the disease.

In the current web implementation of G2D, we free the user from the production of a list of MeSH C terms by requiring instead the identifier of the disease in the OMIM database of human inherited diseases [3] or of a phenotypically equivalent one in that database. For example, a researcher investigating a particular variant of Alzheimer's disease not yet present in OMIM might search the database at the NCBI web server using "Alzheimer" as query term, and use one of the identifiers of the closest variant according to their phenotypes and the user's knowledge. Then, the system compiles automatically a list of MeSH C terms from those present in the MEDLINE references linked to the OMIM entry, weighting them by the fraction of linked MEDLINE references containing them. That is, a MeSH C present in all linked references will be taken more into account than one linked only to one of the references. Currently, a total of 1,663 different OMIM entries that contain enough linked MeSH C terms can be used to query the system.

The chromosomal location is a range that can be defined in three ways: two chromosomal markers (if one is given, a band of 5 Mb is taken around it), two base positions in the build hg17 of the human genome, or two cytogenetic bands (if one is given the band is taken as the range).

The current G2D web server implements new features to further guide the user in the analysis of the results, following strategies that take advantage of the combination of positional data with gene expression data to guide disease-gene data mining [5]. On the one hand, we indicate the overlap of candidate genes with predicted pseudo-genes [6], which suggests that they will not be likely expressed genes. On the other hand, we indicate the overlap of candidate genes with expressed sequence tags (ESTs), which suggests expression of the gene.

## Results

### Benchmark of the method

G2D is a method for the prioritization of genes according to their relation to a disease. The benchmark of such a method must be done testing diseases for which the disease-related gene is known but without providing the system with the obvious link between the target gene and the disease. Otherwise the real capacity of the system in evaluating relations between genes and diseases cannot be assessed. It is also important that the diseases are chosen in a completely unbiased manner.

Therefore, in our previous evaluation of G2D [2], we first compiled a set of 100 monogenic diseases randomly chosen among those with known associated gene according to their entries in the LocusLink database [3]. For the sake of comparison we will use the same set and benchmark procedure in this work, noting that any differences between the two benchmark results will be due exclusively to the newer versions of the databases used, since the method was not changed since then.

In brief, we attempted to remove all experimental and genetic information on the diseases used for the benchmark by producing a version of MEDLINE devoid of references matching the disease names. In this way, we wanted to assure that our algorithm would be discovering the associations of genes to a test-disease based on information extracted from a different disease. Matching entries were found by querying MEDLINE via PubMed using the expansion of the query with synonyms (such as "cancer" and "tumour") and scanning both the abstract of the reference and the MeSH terms associated with it.

For each test-disease, genes in a 30 Mb region centred on the target gene were scored; on average this region contained about 300 genes. This size was taken because such was the average size of the loci of other diseases mapped to a genomic region but for which no gene had been found yet to be related.

The benchmark results were better than those obtained in our previous analysis [2]. The target gene was identified in 87 of the 100 test diseases (55 previously). The target gene was among the 8 best scoring genes in 47 cases (25 previously), and among the 30 best scoring genes in 62 cases (50 previously) (see details of this and previous benchmark in the Supplement page of the G2D web site). The improved system performance was due in part to the improved quality of the hg17 human genome assembly (build 35, supported by the UCSC Genome Bioinformatics Site; May 2004 freeze) [7]. A performance improvement was likely also obtained from the increased number of sequences in RefSeq, and of their functional annotations with Gene Ontology terms (GO terms; [8]), a system for the description of genetic functionality that is also in continuous expansion and refinement.

### Analysis of a monogenic disease: late-onset Alzheimer disease

To illustrate how to use the G2D server for analysis of a monogenic disease, we will be using the recent genetic linkage of a type of late-onset familial Alzheimer disease (LOFAD) to a locus on chromosome 19p13.2 [9] for which no responsible gene has been yet found. Although Alzheimer disease is genetically heterogeneous, there are some common themes among the functions of the proteins related, such as their interaction with the beta-amyloid protein, which can be used to track down a new candidate as we will show.

**Figure 2:**
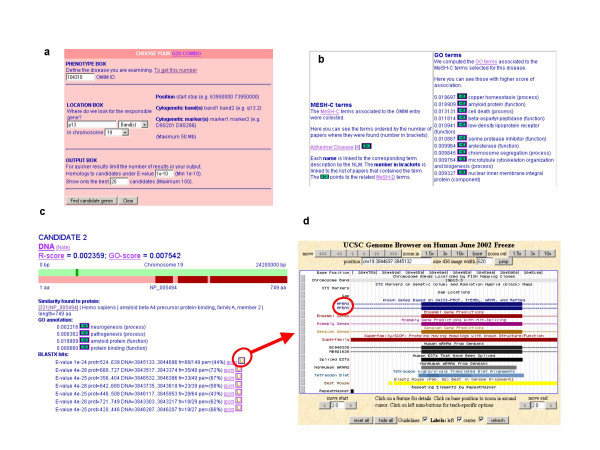
**Example of analysis of a monogenic disease**. (a) The data defining the phenotype of the disease (in this case the OMIM identifier of an equivalent disease) and the region where it was mapped are given in the COMBO box. (b) The results window displays the MeSH C terms derived from the links to MEDLINE found in the OMIM entry, and the resulting scores for the GO terms. The green arrows allow traveling the MeSH C/MeSH D/GO network of connections back and forth. (c) Further down in the results window, the list of candidates displays the position of the BLASTx hits [35] in the chromosomal region (dark green bar over the light green bar) and of the hits in the matching protein sequence (dark red bars over the light red bar). Each hit in the genome is linked to the UCSC Genome Browser ("U" link). (d) The UCSC Genome Browser allows examining the genes known or predicted that overlap with the match linking to very useful databases and resources.

To search for genes that could be associated with the disease in a chromosomal region two inputs are needed: an OMIM identifier of the disease (or of a similar one), and a chromosomal location. The user defines these two inputs in the "COMBO BOX" (see Figure 2a). Following the example, we type in the "PHENOTYPE BOX" OMIM id window, 104310, which is the OMIM id corresponding to another LOFAD, the AD2 that was linked to the gene APOE4 [10]. In the "LOCATION BOX" we define the chromosomal range to be analyzed as band 19p13 on chromosome 19, intentionally wider than the 19p13.2 locus reported for this disease [9]. To do this, we specify that we are defining the location by band by selecting the option "band(s)", and we type the name of the band, p13 in the first window. Finally we select the chromosome where the band is located, 19.

The "OUTPUT BOX" allows changing the restrictions on the number of BLAST hits in the region to be displayed. This is done by modifying two parameters: the maximum BLAST E-value threshold in order to report a hit of sequence similarity in the region to a sequence in RefSeq; and the total number of hits to be displayed, considering that those are sorted by decreasing relatedness to the disease (the derived GO score). More restrictive values (a lower BLAST E-value threshold result in a smaller number of candidates) reduce the time needed for the production and download of the results via a web page, although at the risk of missing an interesting candidate. The default values should be appropriate for a first exploratory search. In this example, we keep the default thresholds (E-value = e-10, and 20 candidates displayed).

The result is a search in a region of 19.8 Mb. The only MeSH C term that is extracted from the MEDLINE links from the OMIM entry, is "Alzheimer Disease" (present in four links). The GO term that receives the highest score is "copper homeostasis", then quite close "amyloid protein". Both are pointed through the MeSH D term "Amyloid beta-Protein Precursor" that is strongly associated with the MeSH C term "Alzheimer Disease". This information can be examined by traveling the network of relations between MeSH C, MeSH D, and GO terms, following the links marked as green arrows (Figure 2b).

The list of candidates contains reports for hits in the 19.8 Mb region to protein sequences in RefSeq (with a BLAST E-value of or below 1e-10). Note that multiple hits may be pointing to the same gene in the region, or that a hit may be pointing to multiple genes in the region (if for example there are two similar genes in the area).

The first hit is given by similarity to a sequence from rat, follistatin, identified by the GO term "serine protease inhibitor", which scores 0.0108. The next one is more appealing, given by similarity to the human amyloid beta A4 precursor protein-binding, which is annotated with the better GO-term "amyloid protein" (Figure 2c). The hits from positions 3,705,192 to 3,701,877 in the negative strand of Chromosome 9, match the N-terminal part (positions 420 to the end, 749) of that protein, which contains a PTB domain (phosphotyrosine-binding domain; positions 367–533) and two PDZ domains (positions 577–655 and 669–735), according to the SMART protein domain search online-tool [11]. PDZ domains are implied in polypeptide binding.

The genomic region of each match can be examined via the UCSC Genome Bioinformatics Site following the links marked with a "U" (see Figure 2d). In this case, the UCSC browser allows confirming that the seven matches of candidate 2 actually overlap with the APBA3 gene that encodes a protein with a similar domain distribution in its C-terminal part, which interacts with the Alzheimer's beta-amyloid precursor protein [12]. As such, this is an ideal candidate to search for mutations in patients of Alzheimer disease.

However, in this particular case, the linkage analysis displayed a maximum of intensity ratio between the markers D19S216 (at position 4.9 Mb) and D19S221 (at position 12.5 Mb), therefore 1.2 Mb further from the telomere than the position of the APBA3 gene, and this led to the group researching this disease to disregard this gene that otherwise they found biologically interesting (Gerard D. Schellenberg, personal communication).

### Analysis of a complex disease: asthma

G2D can be likewise used for complex diseases linked to multiple loci. Users have to repeat the analysis with the phenotype of the disease for each of the linked regions separately. In order to illustrate the analysis of a complex disease, we have pre-computed the analysis of candidate genes for asthma. The results of this analysis are available at the G2D web site. Here, we describe the steps taken to produce this analysis.

Asthma is a common chronic airway disease caused by multigenic influences, many environmental factors, and a high interaction between various risk factors [13]. Disease loci have been mapped to different chromosomes. Although asthma is the complex disease with the largest number of genome scans (currently more than 15 studies) [14], few genes have been associated with the disease (as far as we know; those are ADAM33, DPP10, PHF11 [15], GPRA [16], and Vitamin D receptor [17]). We used the data stored in the Asthma and Allergy Gene Database [18] as an input to G2D to propose candidates in several genomic regions.

To obtain MeSH C terms related to asthma we queried MEDLINE via PubMed with "asthma [tw] AND 'Case Report' [MH]". A total of 4,317 references was retrieved. The most related GO terms were peptidyl-dipeptidase, interleukin-5 receptor, fluid secretion, peroxidase, and protein kinase inhibitor. We then followed up all ten genome scans fully available in the Asthma and Allergy Gene Database [18]. One study provided genome scans from three populations [19] while all others reported one single genome scan [20-26]. Only one trait per study was selected for this analysis (usually asthma except for two studies where total IgE was taken [20,25]). If available, P-values were taken directly from the data files submitted to us. Otherwise LOD scores were converted into P-values by subtracting 1 minus the probability from a chi-squared distribution with 1 degree of freedom of the individual LOD score that has been multiplied before with the 2 fold logarithm of 10 (P = 1 - Pχ^2 ^(2 log10·LOD, 1)). This is a less conservative approximation than suggested elsewhere [27] but used throughout the database. Finally, 233 unique markers with linkage P-values below 0.05 were then selected from these 10 scans. Microsatellite markers were grouped according to recent Marshfield genetic map data. We then defined linkage regions by having at least four supporting markers in a 20 cM interval. This reduced the linkage dataset to 18 regions that were expanded 6 Mb both to the 3' and 5' direction to allow for some imprecision in marker positioning. G2D was used to prioritize genes according to their relation to asthma in each of the regions. The lists of candidate genes obtained for each of the 18 linkage regions are available at the G2D web site.

As a proof-of-principle, we tested the system with one gene recently linked to asthma: ADAM33 in the cytogenetic band 20p13 [28]. It was discovered in a region so far not linked to asthma by at least two different studies and therefore not in our list of target regions. We applied the system to the band defining the disease via OMIM id 600807 ('Asthma susceptibility') and checking the best 3000 scored sequences in RefSeq. ADAM33 was identified (as the 9th best candidate in band 20p13 after removing redundant hits) because of its sequence similarity to ADAM9 encoding for a protein with a disintegrase and metalloproteinase domain 9.

## Discussion

We have implemented in a public web server a method that allows the ranking of genes in a region of the human genome according to their possible relation to a disease. Both the region and the disease can be defined by the user. Since the method is computationally very intensive, mostly due to the amount of genes used for the sequence similarity analysis, we introduced limitations in the maximum size of the genomic region to scan and in the number of candidates to report. Still, the analysis might take a few minutes depending on the load of the server.

We have updated the method and its benchmark with respect to the original version (G2D, [2]) using newer database versions, observing an improvement in performance almost certainly due both to the increased accuracy of the human genome sequence, and to the continuous functional annotation efforts on human sequences and their homologues in other organisms.

In the current version, a test with 100 diseases of known genetic cause indicated that G2D finds the responsible gene in 87 cases out of a pool of 300 genes (on average), the target gene being among the 8 best scoring genes in the 47 of the successful 87 cases.

It must be noted that the identification of candidate genes by G2D relies partly on the sequence similarity comparison of (query) proteins to parts of the genome, and that it is advisable to examine the extent and position of this similarity. For example, the region of similarity could be restricted to a fragment of the query protein not being responsible for the functionality that might be associated to the disease. Moreover, the method could be pointing to a pseudogene. To support the human examination of the results, we indicate the positions of the sequence similarity match both in the query protein and in the genome. We also took advantage of existing information on pseudogene prediction, and we added links to the UCSC genome browser. This allows putting the candidate genes in the context of the latest genetic knowledge, which has been shown to be of great help when identifying genes involved in disease [5].

Although G2D was originally devised for the analysis of single genetic regions, we encourage researchers working on complex diseases to apply the system independently for each of the genomic regions associated with a complex disease (as illustrated in the Results section), provided that the quality of the linkage analysis is good. Actually, it has been shown that when multiple associations of genetic variation to a disease are demonstrated there is a great likelihood that each of them separately constitutes a risk factor contributing to the disease [29]. Accordingly, it is gaining wider acceptance that the classification of a disease as monogenic might be more the result of our lack of knowledge of all the genes involved in that disease than to reality [1,30]. The conceptual separation between monogenic and complex diseases might be illusory.

As far as we know, we have created a unique resource. Other efforts applying data-mining to the study of genes associated with diseases have a different focus. Mainly, none of them uses sequence similarity searches to assess candidate genes so that, in principle, if a disease-related gene lacks functional or protein domain annotation, or if it is not even predicted to be a gene, it will not be detected by such methods.

For example, Freudenberg and Propping [31] also use phenotype information associated with diseases and the GO annotations of genes, but they cluster diseases with similar phenotypes and pool the GO terms of the genes associated. This means that their method cannot distinguish whether a gene will be related to one particular disease but to a pool of diseases. Although the method was tested with a leave-one-out cross-validation in a set of 878 diseases from OMIM with already known associated gene, their results are not as good as ours with 1/3 of cases having the disease-related gene among 160 candidates and the remaining 2/3 being among 1600. However, positional information is not taken into account. The system is not accessible through a web server.

Turner et al. [32] developed POCUS, a method for the prediction of genes related to diseases linked to more than one genomic region. This is exclusively based on the common GO terms and protein domains found among the genes from multiple loci. The performance of the method is shown in a set of 29 diseases and the genes associated with each of them (between three and eleven), by using variable sizes of artificial loci generated around the target genes. Even when using the smallest size (with an average of 20 genes in each loci), the rate of identification of disease genes is of 60 out of 163 disease-genes, with 56 false positives. For the other two larger sizes assayed they find a candidate for only 5 and 4 of the 29 test diseases (using an average of 94, and 187 genes, per loci, respectively) with an increase in the number of false positives. This poor performance in comparison to the methods above is surely due to the fact that they do not use phenotypic information. This, together with the requirement for multiple loci, makes this method complementary to the one above and hard to compare. The method is not available as a web server though some computer programs are given as supplementary material [32].

Finally, we mention the work from van Driel et al. (GeneSeeker, [5]) more focused on the linkage of information related to genes and disease from multiple public databases. GeneSeeker relies on positional information of genetic linkage (to one region), and includes genetic expression information that is extracted for the genes in the region from their entries in sequence databases and MEDLINE references linked therein (but not from ESTs). The method is tested in only ten human malformation syndromes for which the associated gene is known, using an ad-hoc list of organ terms for each one. Obviously, the method would not be able to find a disease-related gene lacking a link indicating expression in an organ. The average of genes in the loci examined was 165. The results vary greatly depending on how the list of terms is applied. In the least restrictive test the gene is found in all the ten cases but among an average of 22 candidate genes. Again, this method is not using any functional information about the genes analyzed or of the disease phenotype, so it is not surprising that its performance is inferior to G2D. Contrary to the methods previously discussed, this method is accessible through a web server.

## Conclusion

Irrespective of the variation between these methods and their complementarity, they represent the effort of the scientific community to put together existing resources for the help of the geneticists searching for disease-related genes. It would be interesting to measure how these efforts are accelerating the pace of disease-related gene discovery. In this respect, we note that at the time we applied G2D for the analysis of a number of monogenic diseases in OMIM for the first time (June, 2001), there was a total of 455 monogenic diseases linked to a genomic region without associated gene. In our second analysis of OMIM that is presented in the G2D web server (July, 2005), the amount of such diseases has risen to 552. In the intervening three years, 104 diseases were associated with genes, representing the successful completion of a gene hunt. However, another 201 genetic diseases were newly mapped to the human genome, leading to a net increase of 97 diseases in our list of analyses. Although the human genome sequence is completed and there is a number of resources available to researchers to assist them in completing the last stages of disease-gene hunt, the bottleneck of the process of characterizing genes associated with diseases is still the definition of the disease-related gene at the end of the search and not the genetic linkage of the disease, or the discovery of new diseases with an inheritance pattern.

Further directions for improvement suggested have been to include other types of information about disease related genes [33], and to take into account the functional links existing between genes associated with a complex disease [32].

Finally, we note that another important factor for improvement is the feedback and collaboration with the experimental groups that are benefited by these new data-mining methods. As with other efforts in Bioinformatics, only by close collaboration between computational and experimental groups can we expect a real advance on the methodology. For this, both parts have heavy duties: computational groups must explain these tools cleanly and clearly, making them openly available in a stable and up-to-date fashion, swift to adapt to the suggestions of the users; but we should not forget that experimental groups must try the tools made for them and in doing that give constructive feedback and fair acknowledgment to their authors.

## Authors' contributions

CP, PB, and MA conceived G2D and associated benchmarks. CP and MA computed the benchmarks, programmed, and maintain the web server. MW contributed to the asthma study. All authors participated in the preparation of the manuscript. All authors have read and approved the manuscript.
